# Real-World Effectiveness of MEpolizuMaB in Eosinophilic Granulomatosis with Polyangiitis in Greece: A Protocol for a Multicentre Retrospective Observational Study - The MEMBER Study

**DOI:** 10.31138/mjr.070825.arn

**Published:** 2026-03-01

**Authors:** Christos Koutsianas, Katerina Antoniou, Dimitrios Boumpas, Theodoros Dimitroulas, Christina G. Katsiari, Menelaos Manoussakis, Kostantinos Porpodis, Kostantinos Samitas, Paschalis Steiropoulos, Paraskevi V. Voulgari, Zafeiris Louvaris, Dimitrios Vassilopoulos

**Affiliations:** 1 Joint Academic Rheumatology Program, Clinical Immunology-Rheumatology Unit, 2^nd^ Department of Medicine and Laboratory, National and Kapodistrian University of Athens, School of Medicine, General Hospital of Athens “Hippokration”, Athens, Greece;; 2 Respiratory Medicine School of Medicine, Laboratory of Molecular and Cellular Pneumonology, School of Medicine, University of Crete, Heraklion, Greece;; 3 Joint Academic Rheumatology Program, 4^th^ Department of Internal Medicine, Attikon University Hospital, National and Kapodistrian University of Athens, School of Medicine, Athens, Greece;; 4 4^th^ Department of Internal Medicine, Hippokration Hospital, Aristotle University of Thessaloniki, Thessaloniki, Greece;; 5 Department of Rheumatology, Faculty of Medicine, School of Health Sciences, University of Thessaly, Larissa, Greece;; 6 Joint Academic Rheumatology Program, Clinical Immunology-Rheumatology Unit, Department of Pathophysiology, School of Medicine, National and Kapodistrian University of Athens, Greece;; 7 Pulmonary Department, Aristotle University of Thessaloniki, G. Papanikolaou Hospital, Thessaloniki, Greece;; 8 7^th^ Respiratory Medicine Dept and Asthma Centre Athens Chest Hospital “Sotiria”, Athens, Greece;; 9 Department of Respiratory Medicine, Medical School, University General Hospital, Democritus University of Thrace, Alexandroupolis, Greece;; 10 Department of Rheumatology, School of Health Sciences, Faculty of Medicine, University of Ioannina, Ioannina, Greece;; 11 Medical Affairs, GSK, Athens, Greece

**Keywords:** EGPA, vasculitides, Mepolizumab (Nucala®), glucocorticoids, remission, interleukin-5

## Abstract

Eosinophilic granulomatosis with polyangiitis (EGPA) is a rare systemic vasculitis occurring predominantly in patients with eosinophilic asthma. Glucocorticoids (GCs) are the cornerstone of treatment for EGPA to achieve disease remission, however, relapses despite GCs therapy are common. Mepolizumab (Nucala^®^) is a humanised monoclonal antibody that binds interleukin (IL)-5 and randomised controlled trials have shown that it effectively targets eosinophil-driven manifestations of EGPA. Since the vasculitic component of EGPA may not be solely driven by eosinophils, the aim of this retrospective observational study is to use real-world evidence to assess clinical outcomes following treatment with mepolizumab (Nucala^®^). A retrospective, observational, cohort study will be conducted in 10 hospital centres specialised in the treatment and monitoring of patients with EGPA in Greece. Data will be retrospectively collected and analysed from medical records of 70 patients with EGPA treated with mepolizumab (Nucala^®^) for ≥ 6 months, covering the period from September, 2021, to December, 2024.

## INTRODUCTION

### Background and rationale

Eosinophilic granulomatosis with polyangiitis (EGPA), formerly known as Churg-Strauss syndrome, is a rare, progressive systemic form of antineutrophil cytoplasmic antibody (ANCA)-associated vasculitis (AAV).^1–3^ EGPA is characterised by a range of clinical features such as eosinophilic asthma, rhinitis or sinusitis, pulmonary infiltrates, neuropathy, and eosinophilic vasculitis targeting one or more end-organs such as heart, lungs, peripheral nerves, skin, gastrointestinal tract and kidneys.^4–7^ Symptomatology of EGPA, includes fever, fatigue, malaise, weight loss, myalgia and arthralgia.^1^ Elevated eosinophils are thought to induce pathogenic effects as a result of tissue and vascular infiltration and inflammation through a variety of mediators.^8,9^

Glucocorticoids (GCs) are the cornerstone of treatment for EGPA to achieve disease remission and reduce symptoms.^3^ Patients with life-threatening manifestations and major organ involvement must receive a combination of GCs and immunosuppressive agents or biologics mainly cyclophosphamide or rituximab, respectively.^3^ However, the majority of patients relapse during the disease course requiring chronic GCs therapy that is associated with increased morbidity and mortality.^10,11^

Mepolizumab (Nucala^®^) is a humanised monoclonal antibody that binds interleukin (IL)-5.^12^ By targeting IL-5, the primary cytokine responsible for promoting eosinophil differentiation, activation, and survival, mepolizumab (Nucala^®^) reduces eosinophil counts within the normal range.^13^ On the basis of the MIRRA study results, mepolizumab (Nucala^®^) was approved in 2017 for the treatment of adult patients with relapsing or refractory EGPA at a dose of 300 mg subcutaneously every four weeks.^12^ The MIRRA study (a 52-week randomised double-blind placebo-controlled phase III study), demonstrated that accrued time in remission (defined as BVAS=0 with a prednisone [or equivalent] dose of ≤ 4 mg/day or > 4.0 mg/day but ≤ 7.5 mg/ day) and proportion of patients achieving remission were significantly higher in the mepolizumab (Nucala^®^) group compared to placebo group. Furthermore, the annualised relapse rate was 1.14 in the mepolizumab (Nucala^®^) group compared to 2.27 in the placebo group (rate ratio, 0.50; 95% CI, 0.36 to 0.70; P<0.001). During weeks 48 through 52 of the study, 44% of the mepolizumab (Nucala^®^) group was able to taper the GCs dose to 4.0 mg or less per day compared to 7% of placebo group.^12^

Real-world data available for mepolizumab (Nucala^®^) in patients with EGPA, derives from studies including small cohorts or subgroup analyses. Their findings support those of the MIRRA study by demonstrating the efficacy and safety of mepolizumab (Nucala^®^), irrespective of EGPA duration, ANCA status, or concurrent use of immunosuppressants.^14–18^

While mepolizumab (Nucala^®^) effectively targets eosinophil-driven manifestations, the vasculitic component of EGPA may not be solely driven by eosinophils. Consequently, it is essential to evaluate the impact of mepolizumab (Nucala^®^) on the progression of disease along with vasculitis-related symptoms in real-world clinical settings for this heterogeneous disease. Indeed, there is limited evidence from real-life cohorts of patients with EGPA where characteristics and outcomes of patients are systematically collected and analysed.

Therefore, the aim of this retrospective observational study is to use real-world evidence to assess clinical outcomes (remission and/ or relapse, disease activity and concomitant use of immunosuppressants) following treatment with mepolizumab (Nucala^®^) in patients with EGPA in Greece.

## STUDY OBJECTIVES

### Primary and Secondary objectives

The primary objective of the study is to assess the frequency of remission among patients with EGPA at 6, 12, 24 and 36 months after the initiation of mepolizumab (Nucala^®^) treatment. Secondary objectives are to assess changes in: *i.* disease severity and *ii.* disease manifestations and organ involvement at 6, 12, 24 and 36 months after the initiation of mepolizumab (Nucala^®^) treatment.

**MATERIALS & METHODS**

### Study design and procedures

The present study is a multicentre, non-interventional, observational retrospective cohort (single arm) study (GSK study ID: 222428) that will be conducted in Greece.

To address the primary and secondary objectives of the study the data will be collected referring to the period between September 1^st^ of 2021 and December 31st of 2024 (retrospective look-back follow up period from mepolizumab (Nucala^®^) initiation). This period was selected in light of European Medicines Agency (EMA) approval of mepolizumab (Nucala^®^) in September 2021 and the need to collect long-term data on the clinical outcomes following mepolizumab (Nucala^®^) treatment in Greek population with EGPA.^19^

The study will be conducted in 10 Greek hospital centres specialised in the treatment and monitoring of patients with EGPA. Specifically, four centres will be from Athens, two from Thessaloniki, and four from major cities throughout Greece (i.e., Heraklion-Crete, Ioannina, Larissa and Alexandroupolis). Therefore, it is expected that the Greek population with EGPA will be adequately represented geographically by selecting these 10 study sites and that the results will be generalisable to the overall Greek population.

The study will be conducted in accordance with ethical principles consistent with the Declaration of Helsinki, Good Pharmacoepidemiology Practices and the applicable legislation on Non-Interventional/Observational Studies. The Ethics Committees/IRBs/IECs of the 10 hospitals have approved the protocol and the patient Informed Consent Form (ICF) for retrospective data use according to local regulations before data collection. The names of the Ethics Committees, approval dates, and protocol numbers, respectively, of the 10 study sites, are listed below: 1. Second Department of Medicine, General Hospital of Athens “Hippokration”, #82, 10^th^ April 2025; 2. Pulmonary Clinic, University General Hospital of Alexandroupolis, #16533, 10^th^ April 2025; 3. Fourth Department of Medicine, General University Hospital of Athens “Attiko”, # n/a, 8^th^ April 2025; 4. Rheumatology clinic, General University Hospital of Ioannina, #269, 28^th^ April 2025; 5. 7th Pulmonology Clinic of General Hospital of Chest Diseases, Athens, “Sotiria”, #84, 29^th^ April 2025; 6. Pulmonology Clinic, General Hospital of Thessaloniki, “G. Papanikolaou”, 341, 8^th^ May 2025; 7. 4th Department of Internal Medicine, Hippokration Hospital, Aristotle University of Thessaloniki, #279, 14^th^ May 2025; 8. Pulmonary Clinic, University General Hospital of Heraklion, #11401, 3rd July 2025; 9. Pathophysiology Clinic, Unit of Autoimmune Rheumatic Diseases and Expertise Centres, University General Hospital of Athens, “Laiko”, #296; 7th October 2025; and 10. Rheumatology and Clinical Immunology” Clinic, University General Hospital of Larissa, #31173, 21st July 2025. Any protocol amendments will be submitted to the same local Ethics Commission and communicated to the clinical trial database for non-interventional retrospective studies (www.dilon.sfee.gr).

Once the patient has been adequately informed and has signed the ICF, their eligibility for the study will be assessed according to the inclusion/ exclusion criteria. The protocol is reported according to Strengthening the Reporting of Observational Studies in Epidemiology (STROBE checklist).

The schedule and assessments that will be performed are presented in **Table 1**. All data in the present study will be retrospectively collected at each hospital centre. For each subject, the data collected will include those available at the time of mepolizumab (Nucala^®^) treatment initiation (i.e., baseline) and at 6±2, 12±2, 24±2, and 36±2 months after the initiation of mepolizumab (Nucala^®^) treatment (**Table 1**). Data collection timepoints are set based on standard EGPA clinical practice, patient follow-up, and the study’s required treatment duration. Nevertheless, due to the nature of a non-interventional retrospective study, a 2-month deviation at each post-mepolizumab (Nucala^®^) data collection timepoint is foreseen.

**Table 1.** Schedule of assessments.

Data Collection timepoints*	**Study entry/Screening**	**Prior to Mepolizumab star**t	**Baseline (Mepolizumab start)**	**After Mepolizumab start, months**							
**6±2**	**12±2**	**24±2**	**36±2**
Assessments							
Informed consent	X						
Eligibility	X						
Demographics			X				
Medical history including detailed information on EGPA history		X	X	X	X	X	X
Blood eosinophil count/μl			X	X	X	X	X
EGPA disease type (relapsing/ refractory/ both)		X					
Evolution of EGPA			X	X	X	X	X
ANCA status			X	X	X	X	X
Mepolizumab treatment history (duration and dose)			X	X	X	X	X
Comorbidities		X	X	X	X	X	X
Concomitant medication(s)		X	X	X	X	X	X
BVAS			X	X	X	X	X
VDI			X	X	X	X	X
Relapse assessment			X	X	X	X	X
ACQ-6			X	X	X	X	X
ACT			X	X	X	X	X
Prednisolone prednisone (or equivalent) dose		X	X	X	X	X	X
Immunosuppressive treatment (medication/ dose)		X	X	X	X	X	X
Spirometry (FEV_1_)			X	X	X	X	X

EGPA: Eosinophilic Granulomatosis with PolyAngiitis; ANCA: AntiNeutrophil Cytoplasmic Antibody: BVAS: Birmingham Vasculitis Activity Score; VDI: Vasculitis Damage Index; ACQ-6: Asthma Control Questionnaire; ACT: Asthma Control Test; FEV_1_: Forced Expiratory Volume in 1 second.

Mepolizumab: (Nucala®).

No diagnostic or therapeutic intervention will be applied outside of standard clinical practice, in the context of this study. Data will be collected if available in the patient’s medical records to maintain the non-interventional approach of the study. In case a patient has not visited the study centre within the defined time points (including the 2-month window for each), i.e. 6±2 months, 12±2 months, 24±2 months, and 36±2 months, meaning no data is available from his/her records at these points, the visit will be considered missed.

### Exclusion Criteria

A patient who meets any of the following criteria will be excluded from participation in the study.

1. Treatment with mepolizumab (Nucala^®^) for EGPA for less than 6 months.
2. Participation in an interventional clinical study during the retrospective look-back follow up period (between 1^st^ September 2021 and 31^st^ December 2024).

### Inclusion Criteria

Patients must meet all the following criteria to be eligible for inclusion in the study:

1. Male or female patient ≥ 18 years old
2. Patient who has been diagnosed with EGPA based on the history or presence of asthma plus eosinophilia (> 1.0x10^9^ /L and/or > 10% of leucocytes) plus at least two of the following additional features of EGPA: a biopsy showing histopathological evidence of eosinophilic vasculitis, or perivascular eosinophilic infiltration, or eosinophil–rich granulomatous inflammation, neuropathy, mono or poly (motor deficit or nerve conduction abnormality), pulmonary infiltrates, non–fixed, sino–nasal abnormality, cardiomyopathy (established by echocardiography or magnetic resonance imaging [MRI]), glomerulonephritis (haematuria, red cell casts, proteinuria), alveolar haemorrhage (by bronchoalveolar lavage), palpable purpura, positive test for ANCA (MPO or PR3).
3. History of relapsing or refractory disease before the initiation of mepolizumab (Nucala^®^) treatment.
4. Patient who has been treated with mepolizumab (Nucala^®^) for EGPA according to local practice.
5. Treatment initiation with mepolizumab (Nucala^®^) 300 mg once every 4 weeks as specified in the approved summary of products characteristics.
6. Provision of a signed and dated ICF.

### Outcome definitions

#### Primary Outcome

The primary outcome will be the proportion of patients in remission at 6, 12, 24, and 36 months after starting mepolizumab (Nucala^®^) based on two definitions derived from the European League against Rheumatism (EULAR) recommendations and the MIRRA study.^3,12^

Remission definition: A: No disease activity according to Birmingham Vasculitis Activity Score (BVAS = 0) and the receipt of prednisone (or equivalent) at a dose of ≤4.0 mg/day.

Remission definition B (less stringent): No disease activity (BVAS = 0) and the receipt of prednisone (or equivalent) at a dose of >4.0 mg/day but ≤7.5 mg/day. BVAS, version 3, will be used to assess systemic disease activity following mepolizumab (Nucala^®^) treatment.^20^

### Secondary Outcomes

The evolution of disease activity will be assessed by the number of relapses measured at 6, 12, 24 and 36 months after starting mepolizumab (Nucala^®^). A relapse of EGPA is defined as worsening in the clinical status of the patient attributed to disease activity. Specifically, active vasculitis (BVAS > 0), or active asthma symptoms or signs with a corresponding worsening in the score on the Asthma Control Questionnaire, version 6 (ACQ-6; range 0 to 6 points, with higher scores indicating worse disease control; minimal clinically important difference, 0.5 points) or worsening of active nasal or sinus disease which required: an escalation in the dose of GCs to more than 4.0 mg/day of prednisolone (or equivalent), or an initiation of or increase in the dose of immunosuppressive treatment, or hospitalisation related to EGPA worsening and as defined in the MIRRA study.^12,21–22^

The time to first relapse of EGPA during mepolizumab (Nucala^®^) treatment will also be recorded. The onset of a relapse will be defined as the time of: an increase in the dose of GCs, or an initiation/increase in the dose of immunosuppressive therapy, and/ or hospitalisation, in association with the worsening in BVAS score, asthma or sino-nasal symptoms.

The evolution of disease severity/damage at 6, 12, 24 and 36 months after starting mepolizumab (Nucala^®^), will be assessed as by the changes from baseline [i.e., before starting mepolizumab (Nucala^®^)] in Vasculitis Damage Index (VDI), Force Expiratory Volume (FEV_1_), Asthma Control Test (ACT). Vasculitis Damage Index (VDI) and the GCs- and immunosuppressants-sparing effect.^12,23^ The VDI covers 11 organ systems and documents damage from vasculitis, treatment, or other causes since disease onset. Damage refers to non-healing scars and does not reflect current disease activity.^23^

The Asthma Control Test (ACT) is a self-administered questionnaire designed to assess how well asthma is controlled over the past four weeks. The ACT score ranges from 5 (poor control) to 25 (complete control), with a score of 20 or higher indicating good asthma control. ^24–26^

The following GC-sparing outcomes will be assessed: *i.* the proportion of patients with ≥ 50% reduction from baseline in dose and use of GCs at 6, 12, 24 and 36 months; *ii.* the proportion of patients with 100% reduction from baseline in dose and use of GCs at 6, 12, 24, and 36 months; *iii.* the proportion of patients receiving daily dose of GCs: 0 mg/day at 6, 12, 24 and 36 months, >0 to ≤4 mg/day at 6, 12, 24, and 36 months, >4 to ≤7.5 mg/day at 6, 12, 24 and 36 months, >7.5 mg/day at 6, 12, 24, and 36 months; and *iv.* the mean GCs daily dose at baseline, 6, 12, 24, and 36 months.

The following immunosuppressant-sparing outcomes will be assessed: *i.* the proportion of patients with ≥ 50% reduction from baseline in the dose of immunosuppressants at 6, 12, 24 and 36 months, and *ii.* the proportion of patients with 100% reduction (complete discontinuation) from baseline of immunosuppressants at 6, 12, 24, and 36 months.

Finally, the changes in disease activity in various organs will be assessed by the BVAS tool. The different EGPA-organ manifestations following mepolizumab (Nucala^®^) treatment will be assessed separately for each BVAS item (BVAS, version 3).^20^ BVAS is a comprehensive multisystem clinical assessment used in therapeutic studies of systemic vasculitis. The BVAS includes scored items grouped into 9 different organ systems (general, cutaneous, mucus membranes/ eyes, ear/nose/throat, chest, cardiovascular, abdominal, renal, nervous system) which capture a broad spectrum of clinical manifestations from vasculitis. The proportion of patients experiencing changes from baseline [i.e., prior mepolizumab (Nucala^®^) treatment] in the 9 BVAS score items separately at 6, 12, 24 and 36 months after the initiation of mepolizumab (Nucala^®^) treatment will be assessed.^1^

### Data sources

Data will be obtained from clinical records and findings, observations, or other sources (e.g., hospital records, clinical and office charts, electronic patient records, laboratory notes) available for each patient at each study site. In the present study, no central electronic database will be used. Data will be collected anonymously in digital formats and will be stored for each centre in an electronic database through eCRF, with password-protected access.

The following characteristics of the patient and parameters will be recorded by the Investigators of each centre: demographics/ anthropometrics, medical history, blood eosinophil count/ μl, history of EGPA [time of diagnosis, disease type (relapsing/ refractory/ both), disease characteristics and treatment received up to initiation of mepolizumab (Nucala^®^)], ANCA status, history of mepolizumab (Nucala^®^) treatment (duration and dose), comorbidities, concomitant treatments, evolution of EGPA, BVAS score, dose and type (medication) of GCs and/or immunosuppressive therapy. In addition, the time and reasons for switching or discontinuation of mepolizumab (Nucala^®^) treatment and, if applicable, the change in treatment will also be recorded (**Table 1**).

Switching or discontinuation of mepolizumab (Nucala^®^)

Discontinuation of mepolizumab (Nucala^®^) and starting another EGPA treatment instead (i.e., another eosinophil-targeted therapy) will be considered a treatment switch. Adding another EGPA treatment (i.e., immunosuppressive treatment) while taking mepolizumab (Nucala^®^) and not instead of mepolizumab (Nucala^®^) will not be considered as a treatment switch. Discontinuation of mepolizumab (Nucala^®^, i.e., 300mg once every 4 weeks) will be defined as more than 12 weeks (or more than 84 days, equivalent) of mepolizumab (Nucala^®^) treatment gap [without mepolizumab (Nucala^®^) treatment]. This means, at least three consecutive missed doses.^26^ In case a participant has switched or discontinued mepolizumab (Nucala^®^), the time to switch or discontinuation and the reasons for the switching or discontinuation and, if applicable, the change in treatment will be recorded.

### Study size

Sample size calculation is based on the expected percentage of patients in remission after treatment with mepolizumab (Nucala^®^) (primary study outcome) from the results of MIRRA study (i.e., a total of 53% of mepolizumab-treated patients achieved remission).^12^ Using the 95% Clopper-Pearson Confidence Interval (CI) and assuming that 50% of patients will be in remission following mepolizumab (Nucala^®^)treatment (i.e., no disease activity BVAS = 0 and the receipt of prednisone or equivalent at a dose of ≤ 7.5 mg/day at any time point 6, 12, 24, or 36 months) the precision to be obtained of 12.20% (95% CI:37.80–62.20) with a sample size of 70 consecutive patients diagnosed with EGPA is adequate to assess the primary outcome of the study. Nevertheless, the achieved precision of percentage of remission will be reported with the final study results.

### Data analysis

Descriptive statistics will be used for the analysis of all primary and secondary outcomes. Continuous variables will be summarised using the number of observations, proportions, mean, median, standard deviation, interquartile range, confidence intervals, minimum and maximum values. Categorical or binary variables will be summarised using the number and percentage of subject in each category. Analysis of the data will be based on scheduled visits/study time points. A disposition summary table with number of patients entered the study, completed the observation period, and discontinued treatment as well as the reasons for discontinuation will be provided. Time to treatment discontinuation will be illustrated by Kaplan Meier curves while the corresponding time periods will be calculated. The proportion of patients who were in remission at each of the following timepoints 6, 12, 24 and 36 months after the initiation of mepolizumab (Nucala^®^) treatment will be provided with the corresponding 95% CI in the overall study population. In addition, the proportion of patients who were in remission at each of the following timepoints 6, 12, 24, and 36 months after the initiation of mepolizumab (Nucala^®^) treatment will be assessed using a generalised linear mixed model for longitudinal binary data using logit transformation. The effects of treatment over time will be shown by the odds of treatment response at 6 months versus 12, 24, and 36 months. The proportion of patients who had at least one relapse at 6, 12, 24 and 36 months after the initiation of mepolizumab (Nucala^®^) treatment will be provided with the corresponding 95% CI in the overall study population based on the observed values. The Kaplan-Meier survival estimate of the time to first relapse along with the corresponding 95% CI will be also provided. The number of patients at risk at each time point as well as the median time to relapse (days) will be also provided. Mixed Models for Repeated Measures (MMRM) will be employed to examine the effects of treatment over time for the secondary outcomes. Confounders and effect modifiers, including demographic and clinical characteristics of the patients, will also be considered in the data analysis.

Summary statistics will be performed only on subjects with available information. The number of missing data will be reported for each measured variable. Unknown or missing data will not be accounted for in computing proportions/ frequencies of categorical variables. Missing data will not be imputed/ extrapolated.

### Data management and sources of bias

A fully validated Electronic Data Capture (EDC) will be used for the present study. Data will be collected in anonymously digital formats (electronic Case Report Form) via a Web-Based Data Capture (WBDC) system ensuring all applicable data protection regulations and requirements with regards to electronic records and database validation. The retrospective design of this study presents potential sources of bias that should be carefully considered when interpreting the results. Reliance on routinely collected clinical data may introduce missing data bias, as variations in accuracy, completeness, and consistency can occur across the participating sites. Additionally, confounding bias cannot be fully excluded. Although multivariable adjustments will be made for confounders such as demographics, EGPA history, and clinical characteristics of the patients, recorded at baseline, the impact of potential unmeasured or residual factors may still influence the findings observed in this study.

### Study status

Data collection is scheduled to begin in June 2025 and is anticipated to conclude in March 2026. Data analysis is expected to be completed by June 2026. The study has been registered in the Greek clinical trial database for non-interventional retrospective studies (www. dilon.sfee.gr, study ID: 248). Results from the study will be presented at scientific meetings as abstracts for poster or oral presentations and published in peer reviewed journals.
